# Repetitive Concussive Traumatic Brain Injury Interacts with Post-Injury Foot Shock Stress to Worsen Social and Depression-Like Behavior in Mice

**DOI:** 10.1371/journal.pone.0074510

**Published:** 2013-09-18

**Authors:** Kristen C. Klemenhagen, Scott P. O’Brien, David L. Brody

**Affiliations:** 1 Department of Neurology, Washington University School of Medicine, St. Louis, Missouri, United States of America; 2 Hope Center for Neurological Disorders, St. Louis, Missouri, United States of America; University of Pittsburgh, United States of America

## Abstract

The debilitating effects of repetitive concussive traumatic brain injury (rcTBI) have been increasingly recognized in both military and civilian populations. rcTBI may result in significant neurological, cognitive, and affective sequelae, and is often followed by physical and/or psychological post-injury stressors that may exacerbate the effects of the injury and prolong the recovery period for injured patients. However, the consequences of post-injury stressors and their subsequent effects on social and emotional behavior in the context of rcTBI have been relatively little studied in animal models. Here, we use a mouse model of rcTBI with two closed-skull blunt impacts 24 hours apart and social and emotional behavior testing to examine the consequences of a stressor (foot shock fear conditioning) following brain injury (rcTBI). rcTBI alone did not affect cued or contextual fear conditioning or extinction compared to uninjured sham animals. In the sucrose preference test, rcTBI animals had decreased preference for sucrose, an anhedonia-like behavior, regardless of whether they experienced foot shock stress or were non-shocked controls. However, rcTBI and post-injury foot shock stress had synergistic effects in tests of social recognition and depression-like behavior. In the social recognition test, animals with both injury and shock were more impaired than either non-shocked injured mice or shocked but uninjured mice. In the tail suspension test, injured mice had increased depression-like behavior compared with uninjured mice, and shock stress worsened the depression-like behavior only in the injured mice with no effect in the uninjured mice. These results provide a model of subtle emotional behavioral deficits after combined concussive brain injury and stress, and may provide a platform for testing treatment and prevention strategies for social behavior deficits and mood disorders that are tailored to patients with traumatic brain injury.

## Introduction

Traumatic brain injury (TBI) is defined as impairment of brain function following mechanical force injury [1]. Of the 3.5 million traumatic brain injury cases each year in the United States [2,3], at least 75% are classified as mild [4], and are predicted to have good cognitive recovery within 3-12 months as measured by current standard neurocognitive assessments [5]. However, in contrast to reports of good cognitive outcomes in TBI patients, the prognosis for long-term affective and social behavior is not as positive. In an Australian study of psychiatric disorders pre- and post TBI, depression was the most common diagnosis within 5 years post-injury, and 72% of the post-injury depression cases were novel, occurring in patients with no retrospective diagnosis of depression before the injury [6]. Depression is the most prevalent mood disorder developed after TBI [7], and may also be more resistant to classical pharmacological treatments than depression in the general uninjured population [8]. Furthermore, although more severe TBI is associated with higher risk for development of a mood disorder after TBI, mild or concussive TBI has also been associated with prolonged post-injury psychiatric illness [9].

Increased risk of suicide has also been associated with TBI, although the exact interplay between TBI, mood disorders, social environment, and suicide remains unclear [10]. A large population-based study conducted in Denmark with follow-up of up to 15 years post-injury found increased risk of suicide in TBI patients, with risk increasing with the severity of the injury [11]. Although the risk was highest in the most severely injured patients, suicide rates were still increased by a factor of two or more in patients with mild concussive traumatic brain injury compared to the general population [11]. TBI has been independently correlated with a variety of interpersonal and social problems, including reports of increased dissatisfaction in marriage for the uninjured partner [12], impaired empathy in social interactions [13], and impaired emotional reactivity and identification of emotions [14]. A study of social function one year after mild TBI in a Moroccan cohort found that 31% reported moderate to severe alteration of family relationships, and 19% reported a significant deterioration of their quality of life [15]. Deficits in interpretation of emotional communication and emotive expression have also been observed in children with mild to severe TBI [16]

Although the majority of single, uncomplicated concussions may be associated with good long-term outcomes, there is growing evidence that repeated concussions may have a cumulative effect, significantly impacting neurological, cognitive, and social/emotional function both acutely and chronically. One well-documented example is the effect of multiple concussive events in chronic traumatic encephalopathy (CTE) patients. CTE is often found in professional contact sport athletes who experience multiple concussive brain injuries, and is associated with cognitive deficits, depression, social instability, and suicidality that can manifest several years after the repetitive brain injury occurs [17,18]. Even in the absence of CTE, professional athletes with repeated concussions have higher risk of cognitive impairment [19] and depression [20]. A 9 year prospective study of retired professional football players found a dose-response relationship between number of concussions and risk of developing depression [21]. Student athletes that sustain 3 or more concussions also report decreased quality of life, including social functioning, compared to those that received 2 or fewer concussions [22].

Post-injury stressors may also contribute to the development of long-term post-concussive symptoms in TBI patients. In a study of time taken to return to combat duty after blast injury, patients were separated into two groups, one requiring an average of 7.6 days for recovery and the other group requiring an average of 24.4 days [23]. One of the best predictors of recovery group membership was the presence or absence of combat stress symptoms, accounting for about 21% of the variance [23].

Animal models of changes in behavioral outcomes after blast-related traumatic brain injury and subsequent stress have recently been developed. Kwon and colleagues examined anxiety (open field, elevated plus maze) and spatial memory (Barnes maze) behavior in rats following a single blast induced TBI followed by two weeks of predator odor and unpredictable stress, and found that stress resulted in only a transient increase in anxiety, whereas blast injury combined with stress resulted in a transient increase in anxiety, longer-lasting impairment in spatial memory, and molecular and cellular markers of cell injury and death in the hippocampus and prefrontal cortex [24]. Elder and colleagues tested rats with 3 blast injuries over 3 days in behavior tests 40 days post-injury and found long-lasting increases in anxiety (elevated zero maze), hyperarousal (acoustic startle), decreased exploration in the presence of predator odor (in an open field), and enhanced single-trial cued, but not contextual fear conditioning [25]. The authors suggested that because the blast injuries were administered under anesthesia, in the absence of a simultaneous psychological stressor, blast-derived mild TBI alone can cause increased anxiety and hyperarousal similar to symptoms seen in PTSD.

While these studies have begun to address the interaction of blast TBI, stress, and emotional behavior, no studies to our knowledge have addressed the effect of post-injury stress on repeated blunt impact closed skull traumatic brain injury. Since depression and psychosocial impairment in the context of blunt impact traumatic brain injury appear to be major contributors to poor long-term outcome and even suicide, it is imperative that we develop valid models that capture some core elements of this complex interaction. Such animal models would be useful to test interventions that may promote long-term recovery in these patients. Here, we combine our murine model of repetitive concussive traumatic brain injury (rcTBI) [26] with the stressor of foot shock fear conditioning and extinction on days 3-7 post-injury, and examine the effects of this moderate stressor on subsequent social, anhedonia, and depression-like behaviors during the following two weeks. We hypothesized that rcTBI would cause deficits in fear conditioning extinction, and that injured mice that underwent foot shock stress would have more profound deficits in performance in the social interaction, sucrose preference, and tail suspension tests compared to injured but non-stressed mice. Contrary to our hypothesis, the rcTBI mice did not show any deficit in cued or contextual fear conditioning or extinction, and mice with rcTBI had decreased sucrose preference regardless of whether they had experienced foot shock stress. However, in agreement with our hypothesis, we found that rcTBI mice had deficits in the social interaction and tail suspension tests compared to uninjured shams, and that shock stressed rcTBI mice had greater impairment in these social interaction and depression tests than the non-stressed rcTBI mice. Further characterization of the interaction of rcTBI and stress and their impact on social and affective function will provide a model that can be used to develop novel therapies for TBI patient populations with urgent unmet social and mood behavioral treatment needs.

## Materials and Methods

### Animals

All experiments were approved by the Washington University Animal Studies Committee, Animal Welfare Assurance # A-3381-01, and conducted in accordance with the NIH Guide for the Care and Use of Laboratory Animals [27]. All surgery was performed under isoflurane anesthesia. Male C57Bl/6J mice were purchased from Jackson Labs (Bar Harbor, ME) at 6 weeks of age. Mice were housed with siblings in groups of 2-5 mice per standard cage with Bed O’ Cobs bedding (Andersons, Inc., Maumee, OH) on a 12 hour light-dark cycle (lights on at 06:00) and allowed to acclimate to the colony for one week before surgery. Behavioral testing began 3 days after the second surgery. Ambient temperature was controlled at 20°C to 22°C and the animals were provided with mouse chow (PicoLab Rodent Diet 20, PMI Nutrition International) and water ad libitum.

### Electromagnetic Closed-Skull Traumatic Brain Injury

Surgical and injury procedures were followed as previously described in Shitaka et al, 2011 [26]. Briefly, mice were anesthetized with 5% isoflurane and placed in a stereotaxic frame with a bite bar and rounded head holders (David Kopf Instruments, Tujunga, CA). Body temperature was controlled at 37°C using a feedback temperature controller (Cell Microcontrols, Norfolk, VA), and anesthesia was maintained by isoflurane via nose cone at 1.5-2% in air. The head was shaved, swabbed with Betadine, and a midline skin incision was made to expose the skull.

A rubber tip (Precision Associates, Inc., Minneapolis, MN) 9mm in diameter and with a spring constant of 3.01 N/mm was mounted on an electromagnetic stereotaxic impact device, and the tip was lowered at a 20° angle with the vertex touching the skull at 1.8mm caudal to bregma and 3.0mm to the left of midline. The tip was retracted, lowered 3.3mm, and then triggered, driving the tip into the skull at a depth of 3.3mm at 5.0m/s with a dwell time of 100ms. Deformation of the rubber tip spread the impact force over the skull.

After impact, the incision was closed with two 7 mm surgical staples (Reflex 7, CellPoint Scientific, Inc., Gaithersburg, MD) and swabbed with antibiotic ointment (Neosporin, New Brunswick, NJ). Animals were allowed to recover on a warming pad, and were returned to their home cages when normal ambulation and grooming behavior had resumed. After 24 ± 1 hour, a second identical closed-skull TBI procedure was performed. For sham injuries, the same procedure (including handling and anesthesia duration) was followed, except the impact device was discharged into the air. Within each cage, animals were randomly assigned to the injury or sham group, resulting in both injured and sham animals in each cage. Four to five water-moistened chow pellets were placed on the floor of the cage in a heavy 4.5cm glass tea light holder cup (Target, Minneapolis, MN) on both days following surgery to support recovery of eating and drinking behavior.

Most mice survived the procedure and were returned to their home cages within 15 minutes. In the cued conditioning group, one animal died from apnea during anesthesia, and one from skull fracture. In the contextual conditioning group, two animals died from apnea during anesthesia. Animals with small visible hemorrhages were immediately sacrificed (two in the cued group and two in the contextual group). Final group sample sizes were between 11-14 mice per group (Table 1).

**Table 1:** Experimental groups.

		**rcTBI**	**uninjured sham**
**cued**	shocked	rcTBI, shocked (n=13)	uninjured sham, shocked (n=14)
	not shocked	rcTBI, not shocked (n=13)	uninjured sham, not shocked (n=14)
**contextual**	shocked	rcTBI, shocked (n=12)	uninjured sham, shocked (n=15)
	not shocked	rcTBI, not shocked (n=11)	uninjured sham, not shocked (n=14)

Each experimental group consisted of rcTBI injured and sham uninjured mice, and shocked and not shocked mice, resulting in four groups per experiment. There were two separate experiments, one involving cued fear conditioning, and one that involved contextual fear conditioning. Thus, there were eight groups of mice total. Mice that experienced cued fear conditioning were subsequently tested in the tail suspension and social recognition tests. Mice that experienced contextual fear conditioning were subsequently tested in tail suspension, social recognition and sucrose preference tests.

### Behavior Testing

**Figure 1:**
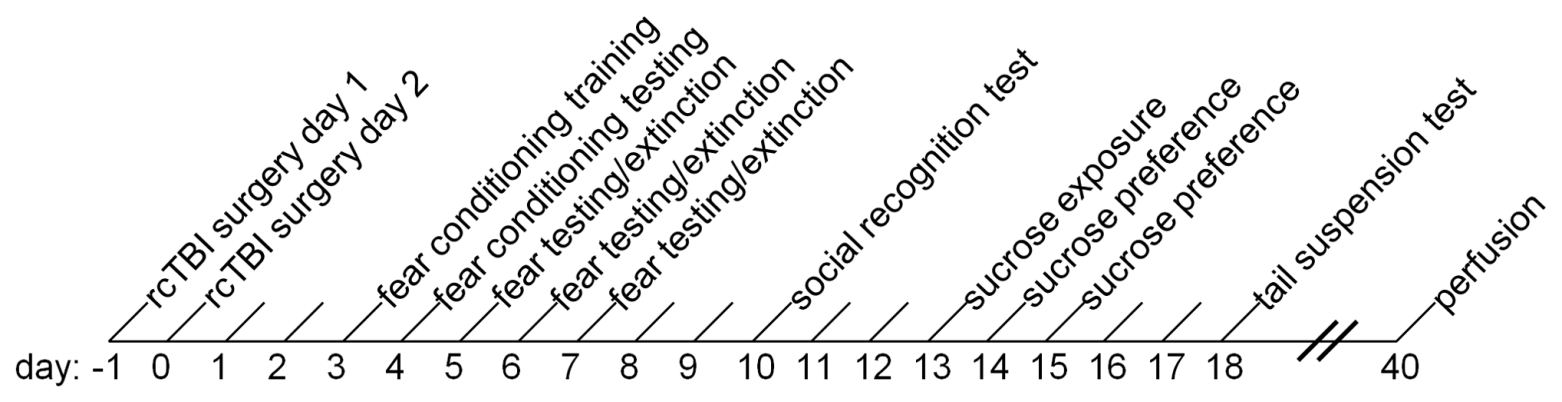
Experimental timeline. rcTBI injury or sham-injury surgeries were carried out on two consecutive days, 24 ± 1 hours apart. After two days of recovery, animals were trained in cued or contextual fear conditioning. Animals were subsequently tested in the social recognition test (day 10), sucrose preference test (contextual fear conditioning group only; days 13-15), and the tail suspension test (day 18). On day 40 post-surgery, animals were sacrificed and perfused for histology.

Fear conditioning was carried out during the light cycle; all other behavior tests were carried out during the dark cycle. Separate groups of animals were tested in either cued fear conditioning or contextual fear conditioning, and then subsequently in social interaction and tail suspension tests. Animals in the contextual fear conditioning group were also tested in the sucrose preference test (see Figure 1 for timing and order of tests). Fear conditioning was carried out by an experimenter blinded to the injury status of the animal, and all other tests were carried out by an experimenter blinded to the injury and shock stress status of the animal.

All fear conditioning and extinction occurred in a chamber (17cm width x 17cm depth x 25cm height) enclosed in a sound-attenuating box (Stoelting Co., Wood Dale. IL). The walls were constructed of clear Plexiglas, and the flooring was constructed of stainless steel rods 1 mm in diameter spaced 5 mm apart connected to a constant current shock source to deliver foot shock. The attenuating chamber was illuminated with a single house light on the ceiling (20 lux). Fresh odorants were pipetted on paper towels placed in the steel pan below the behavioral chamber for each trial. Two different groups of environmental cues were used to establish two unique contexts. Context A consisted of exposed metal bars on the floor of the chamber, a black and white checkered pattern on 3 walls and clear plastic on 1 wall, a white noise fan on (50 dB), lemon extract (McCormick and Co., Hunt Valley, MD) on paper towels below the chamber, and 1% bleach as the cleaner between animals. Context B consisted of a smooth white plastic chamber floor, a striped pattern on 3 walls and solid gray pattern on 1 wall, fan off, peppermint extract (McCormick and Co., Hunt Valley, MD) on paper towels below the chamber, and 70% ethanol as the cleaner between animals. Sessions were digitally video recorded with the software ANY-Maze (Stoelting Co., Wood Dale, IL) at medium video quality and 10 positions/second for later automated freezing analysis.

#### Cued Fear Conditioning and Extinction (post-surgery days 3-7)

The cued fear conditioning protocol was carried out over 5 days. Group sizes were n=13 (rcTBI, shocked), n=13 (rcTBI, not shocked), n=14 (uninjured sham, shocked) and n=14 (uninjured sham, not shocked). On the training day, the animals were placed in Context A (training) and received a conditioned stimulus (tone: 2000Hz, 80 dB, 30 sec duration) along with an aversive unconditioned stimulus (foot shock: 0.4mA, 0.5 sec duration, co-terminated with the tone). Four tone-shock pairings were presented at unpredictable intervals during the 10 minute training session. Non-shock control animals received the same tones but with no foot shock. On testing days 1-4, the animals were placed in Context B (novel), and 5 tones were presented (2000Hz, 80 dB, 30 sec duration) at unpredictable intervals during the 9 minute session. Freezing before the first tone was assessed as a baseline fear measure, freezing during the tones on testing day 1 was assessed as a measure of cued fear conditioning, and freezing during the tones across days 1-4 was assessed as a measure of cued fear extinction.

#### Contextual Fear Conditioning and Extinction (post-surgery days 3-7)

The contextual fear conditioning protocol was carried out over 5 days in separate group of mice. Group sizes were n=12 (rcTBI, shocked), n=11 (rcTBI, not shocked), n=15 (uninjured sham, shocked) and n=14 (uninjured sham, not shocked). On the training day, the animals were placed in Context A (training), where they received an aversive unconditioned stimulus (foot shock: 0.7mA, 0.5 sec duration). Four foot shocks were presented at unpredictable intervals during the 10 minute training session. Non-shock control animals were placed in Context A for 10 minutes on the training day but received no foot shocks. On testing day 1, animals were placed in Context B (novel) for 5 minutes, and then approximately 3 hours later, placed in Context A (training) for 5 minutes. On days 2-4, animals were placed in Context A (training) for 5 minutes. In the contextual fear conditioning group, Context B was made more distinct by additional changed cues of different gloves worn by the experimenter (Context A: beige latex; Context B: blue nitrile), different holding cages prior to testing (Context A: standard cage; Context B: large paper bucket), and different shape of the chamber (Context A: square; Context B: plastic insert to make it circular). Freezing in Context B (novel) was assessed as generalization of fear to a similar context, freezing during the session in Context A (training) on testing day 1 was assessed as a measure of contextual fear conditioning, and freezing during the session in Context A across testing days 1-4 was assessed as a measure of contextual fear extinction.

#### Social Recognition Test (post-surgery day 10)

The social recognition test was adapted from [28] and [29]. Test and stimulus mice were individually housed in standard cages, with bedding and a filter cage top, and without a wire food hopper, and acclimated for at least one hour to the testing room. During testing, an unfamiliar male mouse was used as a stimulus “object”, and was placed in the cage of the test mouse for nine 1-minute interaction/habituation sessions, again without the food hopper and with the plastic filter top. Both the test and stimulus mouse were allowed to move around the cage freely during testing. Stimulus mice were 7-10 week old male C57Bl/6J mice (Jackson Labs) that were housed in the same colony room as the test mice, but had no prior direct contact with test mice and no prior handling other than routine cage changes. The same stimulus mouse was placed in the cage of the test mouse repeatedly for sessions 1-9, and then in session 10 a different novel stimulus mouse was placed in the cage. Individual stimulus mice were used for either habituation or novel sessions, and were used as partners for a maximum of 4 test mice per day. Mice were tested in groups of 5, so each 1-minute interaction session was separated by approximately 5 minutes rest (4 minutes of testing other mice in the group and 1 minute moving stimulus mice in and out of test cages). When there were fewer than 5 mice in a testing group, the experimenter waited between sessions so that the time between sessions was held constant at 5 minutes.

Social interaction was scored by an investigator blind to the injury and prior shock stress status of the test mice. A stopwatch was used to measure the time spent in active social contact performed by the test mouse. Behaviors that were scored as interaction were sniffing with the nose within 1cm of the stimulus mouse (including nose, body, and anogenital area), pawing and climbing on the stimulus mouse, and close following of the stimulus mouse within 2 cm. Social contact initiated by the stimulus mouse was not included in the interaction time measure. Juvenile male mice are often used as stimulus animals in social tests to decrease the incidence of aggression [30]; however, in this case the exposure time of 1 minute was short enough that aggression was not observed in any of the trials.

#### Tail Suspension Test (post-surgery day 18)

The tail suspension test was adapted from [31] and [32]. Mice were suspended by the tail from a horizontal rod 30 cm above the bench surface using adhesive tape. The test was 6 minutes in duration. Since C57Bl/6J mice have been frequently observed to climb their tails during the tail suspension test [33], the tail of each mouse was passed through a cardstock paper cone before attaching the tip of the tail to the rod to prevent climbing behaviors [34]. The cones were 4.5 cm in diameter at the base and 5.5 cm tall, and a new cone was used for each animal. Using this method, no trials were discarded for climbing behavior. Immobility time, defined as motionlessness other than momentum from a prior bout of mobility [32], was scored by an observer blind to the injury and prior shock stress of the animal.

#### Sucrose Preference Test (post-surgery days 13-15)

A 24 hour 2-bottle sucrose/water preference test was conducted according to Strekalova and Steinbusch, 2010 [35]. Testing was initiated at the beginning of the dark cycle. To prevent bias from preference for bottle location, the position of the bottles was switched at the midpoint of testing with respect to light/dark cycle. On the testing day, mice were individually housed at 12: 00, the drinking test began at 18: 00, the bottle positions were switched at 24: 00, 06:00, and 12: 00, and test ended at 18: 00. To minimize error from dripping liquid, the drinking bottles were filled with tap water or 1% sucrose in tap water one day in advance and kept in the upside-down position in the testing room to allow them to equilibrate to room temperature and decrease dripping due to pressure from temperature differences inside and outside the bottle. Drinking bottles were constructed from standard 250 ml mouse drinking bottles and caps (bottle TC15-17BHT, Allentown, Inc., Allentown, NJ) and spring-loaded sipper tubes (taken from Best Buy Pet Bottle, item #03332, Ware Mfg., Inc, Phoenix, AZ). To decrease variability due to neophobia to sucrose, mice were exposed to a single bottle of 2.5% sucrose solution for 6 hours while still group housed one day before testing during the beginning of the dark cycle. Normal chow was available ad libitum before and during the 24 hour sucrose preference test.

Percentage of sucrose preference was calculated as the percentage of 1% sucrose consumed compared to the total volume of water and sucrose consumed (sucrose preference = volume sucrose/(volume sucrose + volume water) x 100).

### Histopathology

A random subset of animals (five each from the rcTBI, shocked, rcTBI, not shocked, uninjured sham, shocked, and uninjured sham, not shocked groups) from the cued and contextual fear conditioning behavioral groups were examined at 40 days post-injury. A previous study of histopathological changes in rcTBI mice indicated steady levels of Iba1 staining at 28 and 49 days post-injury [26], so we hypothesized that sham and injured mice would have comparable differences in Iba1 staining at 40 days post-injury. Random selection of subjects for histological analysis was carried out using the list randomizer at http://www.random.org (Randomness and Integrity Services Limited, Dublin, Ireland).

Animals were deeply anesthetized with an overdose of isoflurane and transcardially perfused with 0.3% heparin in phosphate buffered saline. Brains were removed from the skull, examined for subdural or subarachnoid hemorrhage. No additional hemorrhages were observed. Following removal from the skull, brains were fixed in 4% paraformaldehyde for 24 hours and equilibrated in 30% sucrose for at least 3 days. A freezing sliding microtome (Microm HM 430, Thermo, Fisher Scientific) was used to slice the brain in to 50µm coronal sections from the anterior corpus callosum through the posterior hippocampus.

Iba1 immunohistochemistry was performed using a rabbit polyclonal anti-Iba1 antibody (Wako Chemicals USA, Richmond, VA). Free-floating sections were washed in Tris-buffered saline before blocking with 3% normal goat serum (NGS) in Tris-buffered saline containing 0.25% Triton X (TBS-X) for 30 minutes. Sections were incubated overnight in TBS-X and anti-Iba1 antibody (0.5µg/ml) and 1% NGS. Sections were then washed with TBS and incubated with biotinylated goat-anti-rabbit secondary antibody for 1 hour. Sections were washed with TBS again before detection and visualization with horseradish peroxidase (ABC Elite Kit, PK6100, Vector Laboratories, Burlingame, CA) and DAB (Sigma-Aldrich USA). The 3, 3’ diaminobenzidine (DAB) chromogen also labels red blood cells, and the presence of DAB-labeled blood cells was also used to assess for intraparenchymal hemorrhage. No hemorrhages were observed in the subset of brains used for histological analysis.

### Stereology

Iba1 stereology was performed as described in Shitaka et al. [26]. The optical fractionator probe of StereoInvestigator (Microbrightfield, Williston, VT) was used to count the number of Iba1 positive cells. The grid size was 180μm x 180μm and the counting frame was 80μm x 80 μm. Three sections were quantified per animal, separated by 300μm, ranging from approximately bregma -1.1 to -2.2 mm.

### Statistical Methods

Statistica 6.0 (StatSoft, Inc., Tulsa OK) and GraphPad Prism 5.0 (GraphPad Software, Inc., La Jolla, CA) were used to analyze the data. All data passed the D’Agostino and Pearson omnibus normality test and were tested for homogeneity of variance with the Cochran C, Hartley, and Bartlett test. For repeated measures, the assumption of sphericity was tested via the Mauchley test, and the Geisser-Greenhouse correction to the degrees of freedom and p values is reported when sphericity was violated.

An alpha level of 0.05 was used for all statistical significance tests, with the exception of the main outcome of contextual fear conditioning extinction in the contextual fear conditioning group. In this group, 6 injured animals died unexpectedly on the second day after surgery, during the recovery/rest period and before behavior testing began, leaving sample sizes of 11-12 in the rcTBI injured groups and 14-15 in the uninjured sham groups. Since this decreased the number of subjects in this study below the planned sample size, we chose an alpha level prior to analysis of the contextual conditioning data based on an alpha spending function for information time point 0.80 (80% of planned data collected) using the WinLD group sequential boundary software available at http://www.biostat.wisc.edu/Software/landemets/index.html [36]. We calculated a two-sided symmetric boundary to hold the overall alpha at 0.05 using the Pocock function, resulting in an alpha level of 0.043 for the interim analysis. The alpha value for significance for analysis after increasing the number of subjects to the planned sample size would have been 0.024; however, we chose to stop collecting data because the interim analysis clearly indicated no significant differences between injury and sham groups.

Fear conditioning data were analyzed with three-way repeated measures ANOVA with two between-subjects predictors (injury status: rcTBI or sham; shock status: shocked or not shocked) and one repeated within-subjects factor (freezing across testing days 1-4: time). No significant violations of the assumption of homogeneity of variance were noted for any of the cued or contextual conditioning freezing measures with the single exception of contextual conditioning day 3, minute 4 (p=0.005).

Social Recognition Test data were analyzed with three-way repeated measures ANOVA with injury status and shock status as between-subjects predictors and interaction across habituation sessions 1-9 (time) as a repeated within-subjects factor. A single session in the contextual conditioning group violated the assumption of homogeneity of variance (habituation session 6, p=0.03). Planned t-test comparisons were carried out for the single novel interaction session for rcTBI, shocked vs. rcTBI, not shocked; sham, shocked vs. sham, not shocked; and rcTBI, shocked vs. sham, shocked.

The Tail Suspension Test and Sucrose Preference Test data were analyzed with two-way ANOVAs, with injury status and shock status as between-subjects predictors for the dependent measures (immobility time and percent sucrose preference, respectively).

## Results

### Data Availability

Data from this study have been made publicly available at Dryad (http://datadryad.org). The DOI for the data package is doi: 10.5061/dryad.v1t54 [37].

### rcTBI Does Not Affect Cued Fear Conditioning or Extinction

**Figure 2:**
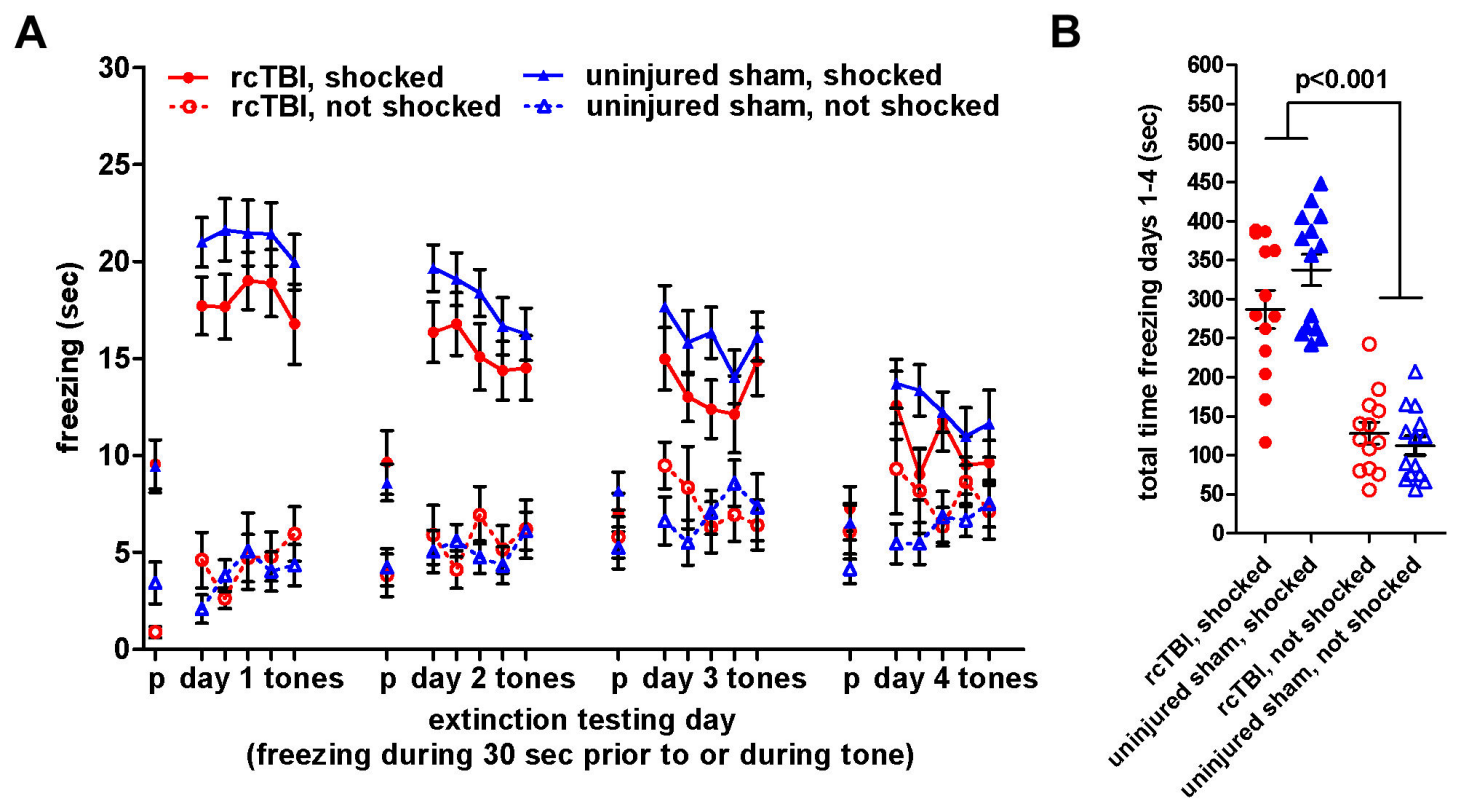
Cued fear conditioning and extinction. Error bars represent SEM. A) Freezing prior to (“p” tick mark) and during the 5 conditioned tones per day on testing days 1-4. Each point represents freezing during a 30 sec. period. B) Total freezing during tones on testing days 1-4 during a total of 20 tone presentations. 2-way ANOVA shows a main effect of shock (p<0.001), but no main effect of injury (p=0.349). Bonferroni post-hoc testing shows no significant difference between rcTBI, shocked and sham, shocked (p=0.340, n.s.), or rcTBI not shocked and uninjured sham, not shocked (p=1.000, n.s.).

Freezing behavior to the aversively conditioned stimulus tone was measured during five 30-second tone presentations on each of 4 days of testing (Figure 2). Three-way repeated measures ANOVA showed main effects of shock [F(1, 50)=110.2, p<0.001] and time [F(11.9, 593.0)=4.2, p<0.001], but no main effect of injury [F(1, 50)=0.9, p=0.349]. There was a significant interaction between time and shock [F(11.9, 593.0)=16.3, p<0.001], but not between time and injury [F(11.9, 593.0)=0.7, p=0.747] or shock and injury [F(1, 50)=3.3, p=0.076]. Both rcTBI and sham animals learned the association between the tone and foot shock, and did not differ in their extinction of the learned fear response over time. There was no difference in freezing between the rcTBI and sham not shocked controls, suggesting that rcTBI does not affect baseline freezing levels.

Freezing was also measured during a 30 second period before the presentation of the first tone on each testing day as a baseline measure of freezing behavior. Three-way repeated measures ANOVA showed a significant main effect of shock [F(1, 50)=25.76, p<0.001], but no main effect of injury [F(1, 50)=0.0, p=0.963] or time [F(2.5, 123.8)=0.78, p=0.485]. Despite the tones being presented in a novel context on the testing days, both rcTBI and sham animals that received shock generalized their fear to the similar context to some extent, shown in the increased freezing in the shocked vs. not shocked control groups. However, they did not differ in the amount of generalization or the expression of generalized fear across time. The rcTBI, not shocked and sham, not shocked animals also did not significantly differ from each other in these baseline measures, again suggesting that rcTBI does not affect baseline freezing.

### rcTBI Does Not Affect Contextual Fear Conditioning or Extinction

**Figure 3:**
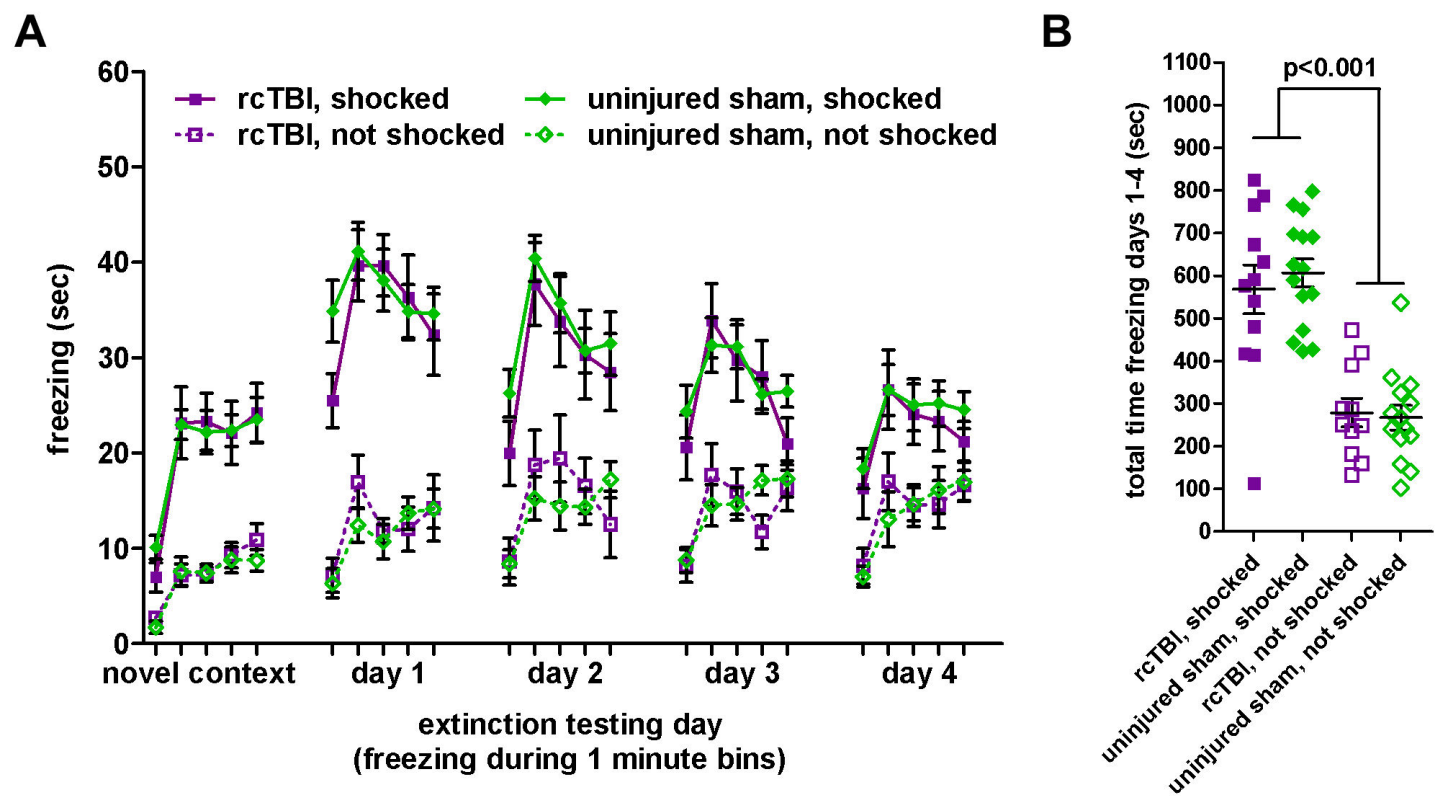
Contextual fear conditioning and extinction. Error bars represent SEM. A) Freezing separated into 5 one-minute bins in a novel context, and in the training context on days 1-4. B) Total freezing in the training context on days 1-4 during a total of 20 minutes of testing. 2-way ANOVA shows a main effect of shock (p<0.001), but no main effect of injury (p=0.727). Bonferroni post-hoc testing shows no significant difference between rcTBI, shocked and sham, shocked (p=1.000, n.s.), or rcTBI, not shocked and sham, not shocked (p= 1.000, n.s.).

In separate groups of mice, freezing was first measured in a novel context for 5 minutes in 1-minute bins as a baseline measure of freezing behavior (Figure 3). Three-way repeated measures ANOVA showed significant main effects of shock [F(1, 48)=74.50, p<0.001] and time [F(3.8, 182.1)=44.0, p<0.001], but no main effect of injury [F(1, 48)=0.01, p=0.916]. Despite efforts to make this novel context as different as possible from the training context, both rcTBI and sham animals that received shock generalized their fear to the novel context to some extent, shown in the increased freezing in the shocked vs. the not shocked control groups. In this case, shocked and not shocked animals did differ in their behavior across the session, but the rcTBI and sham animals did not significantly differ from each other in these baseline measures.

Freezing behavior to the aversively conditioned environment was then measured during 5 minute testing trials divided into 1-minute bins on each of 4 days of testing. Three-way repeated measures ANOVA showed main effects of shock [F(1, 48)=66.1, p<0.001] and time [F(9.2, 441.4)=15.7, p<0.001], but no main effect of injury [F(1, 48)=0.12, p=0.729]. There was a significant interaction between time and shock [F(9.2, 441.4)=9.22, p<0.001], but not between time and injury [F(9.2, 441.4)=1.06, p=0.392] or shock and injury [F(1,48)=0.42, p=0.520]. Both rcTBI and sham animals learned the association between the environment and foot shock, and did not differ in their extinction of the learned fear response over time. There was no difference in freezing between the rcTBI and sham not shocked controls, repeating the finding from the independent cued conditioning group that rcTBI does not affect baseline freezing levels.

### rcTBI Combined with Prior Shock Stress Reduces Social Interaction in the Social Recognition Test in the Cued Conditioned Group

**Figure 4:**
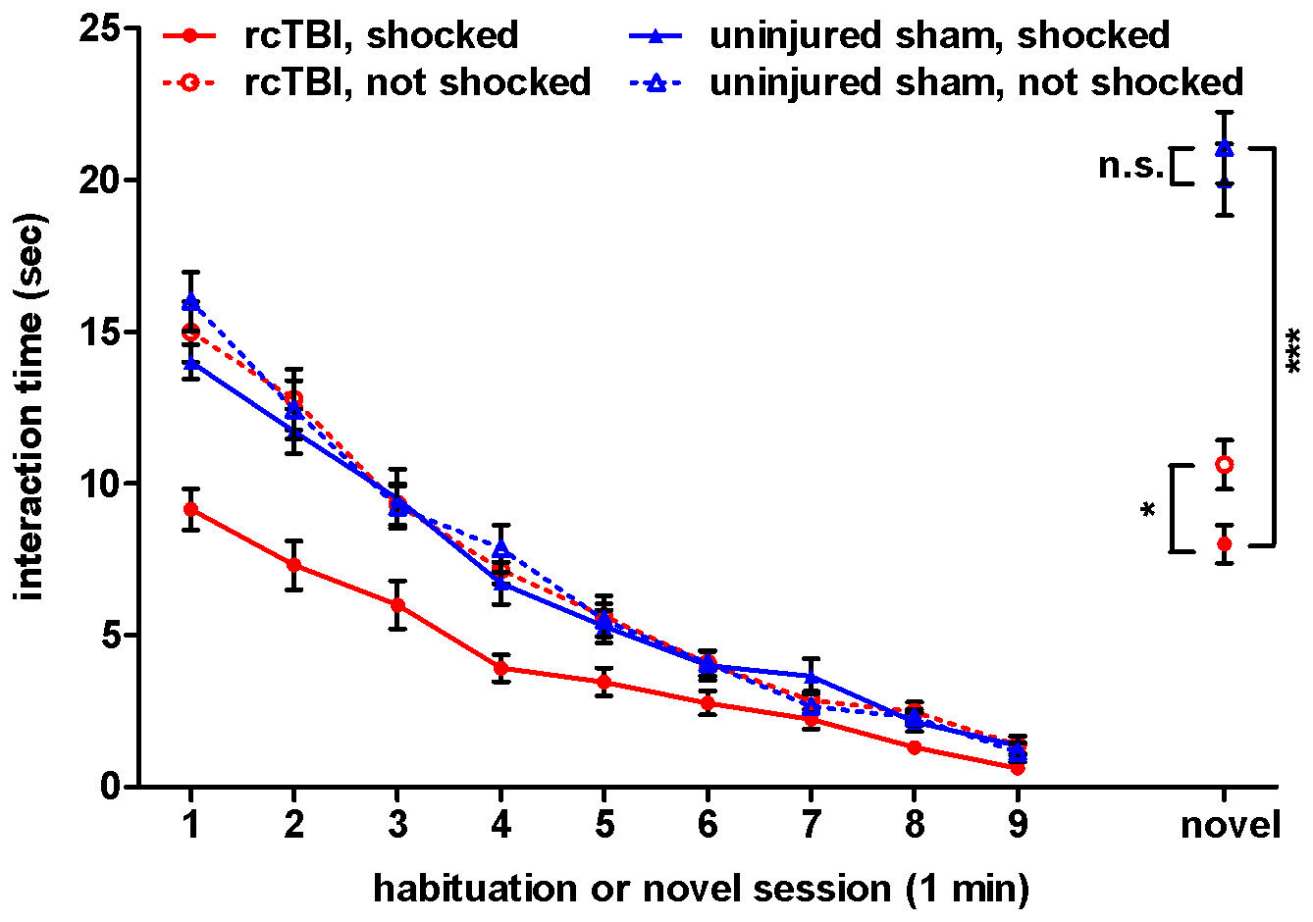
Social recognition test with previously cued fear conditioned mice. Time spent interacting with a stimulus mouse over 9 one-minute habituation sessions and a one-minute novel stimulus mouse session. Three-way ANOVA shows significant main effects of injury (p=0.004), shock (p=0.001), and time (p=0.000) during habituation. In the novel stimulus session, planned comparison t-tests show significant differences between rcTBI, shocked and rcTBI, not shocked (p=0.017, *); rcTBI, shocked and sham, shocked (p=0.000, ***); but not between sham, shocked and sham, not shocked (p=0.527, n.s.). Error bars represent SEM.

Initial social interaction with a novel stimulus mouse and habituation of social interaction with the same stimulus mouse were measured over 9 interaction sessions of 1 minute each (Figure 4). In a three-way repeated measures ANOVA, there were significant main effects of injury [F(1, 50)=9.0, p=0.004], shock [F(1, 50)=13.1, p=0.001], and time [F(3.3, 164.9)=323.3, p<0.001]. There were also significant interactions between injury and shock [F(1, 50)=8.2, p=0.006], time and injury [F(3.3, 164.9)=3.7, p=0.010], and time and shock [F(3.3, 164.9)=8.2, p<0.001]. However, there was not a significant three-way interaction between time, injury, and shock [F(3.3, 164.9)=2.1, p=0.093].

We analyzed the final interaction session with the second novel stimulus mouse as a separate sub-experiment and carried out three planned comparison t-tests between groups. In an initial two-way ANOVA analysis of this session, there was a significant main effect of injury [F(1, 50)=126.5, p<0.001], but no significant main effect of shock [F(1, 50)=3.4, p=0.071] or interaction between injury and shock [F(1, 50)=0.60, p=0.443]. In planned comparison t-tests, there was a significant difference in social interaction shown by the test mice toward the novel mouse in the rcTBI, shocked vs. rcTBI, not shocked groups (t=2.56, p=0.017) and the rcTBI, shocked vs. sham, shocked groups (t=8.736, p<0.001). However, there was no significant difference between the sham, shocked vs. sham, not shocked groups (t=0.64, p=0.527).

### rcTBI Combined with Prior Shock Stress Reduces Social Interaction in the Social Recognition Test in the Context Conditioned Group

**Figure 5:**
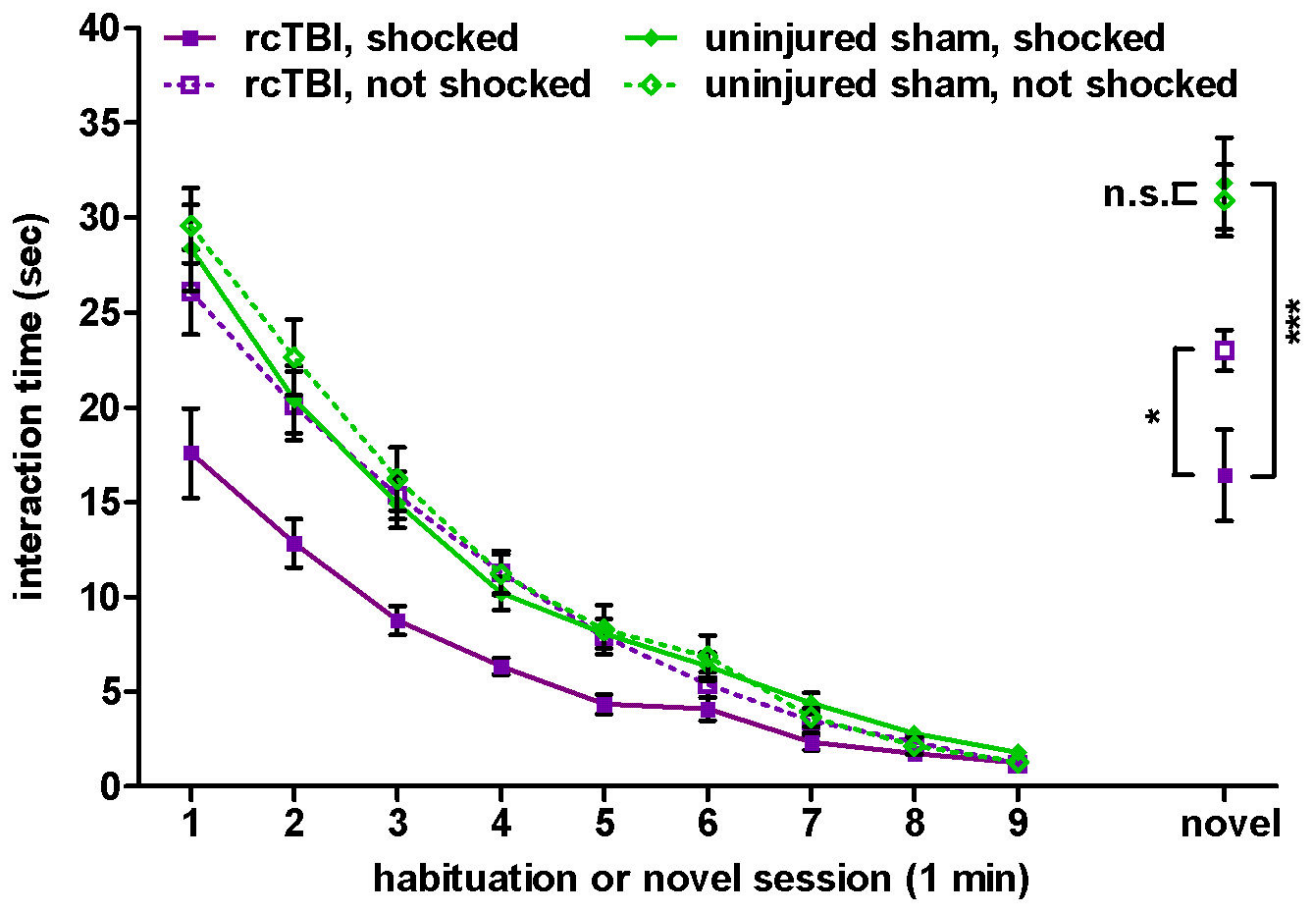
Social recognition test with previously context fear conditioned mice. Time spent interacting with a stimulus mouse over 9 one-minute habituation sessions and a one-minute novel stimulus mouse session. Three-way ANOVA shows significant main effects of injury (p=0.001), shock (p=0.007), and time (p=0.000) during habituation. In the novel stimulus session, planned comparison t-tests show significant differences between rcTBI, shocked and rcTBI, not shocked (p=0.025, *); rcTBI, shocked and sham, shocked (p<0.001, ***); but not between sham, shocked and sham, not shocked (p=0.780, n.s.). Error bars represent SEM.

The same analysis was applied to this group of animals as was carried out for the cued conditioning group reported above (Figure 5). In a three-way repeated measures ANOVA of the habituation interaction sessions, there were significant main effects of injury [F(1, 48)=11.7, p=0.001], shock [F=(1, 48)=7.8, p=0.007], and time [F(2.4, 114.3)=274.9, p<0.001]. There were also significant interactions between injury and shock [F(1, 48)=4.6, p=0.038], time and injury [F(2.4, 114.3)=5.3, p=0.004], and time and shock [F(2.4, 114.3)=4.4, p=0.010]. There was no significant three-way interaction between time, injury, and shock [F(2.4, 114.3)=1.4, p=0.243].

The final interaction session with the novel stimulus mouse was again analyzed with three planned comparison t-tests. Initial two-way ANOVA analysis of this session revealed a significant main effect of injury [F(1,48)=30.3, p<0.001], but no significant main effect of stress [F(1,48)=1.8, p=0.184] or interaction between injury and stress [F(1,48)=3.1, p=0.085]. In planned comparison t-tests, there was a significant difference in social interaction shown by the test mouse toward the novel mouse in the rcTBI, shocked vs. rcTBI, not shocked groups (t=2.42, p=0.025) and the rcTBI, shocked vs. sham, shocked groups (t=4.462, p<0.001). However, there was no significant difference between the sham, shocked vs. sham, not shocked groups (t=0.282, p=0.780). These results independently replicate the findings with the previous group, which received cued fear conditioning prior to this test instead of contextual fear conditioning.

### rcTBI Increases Immobility in the Tail Suspension Test, and This Deficit is Exacerbated by Prior Shock Stress in Both the Cued and Context Conditioned Groups

**Figure 6:**
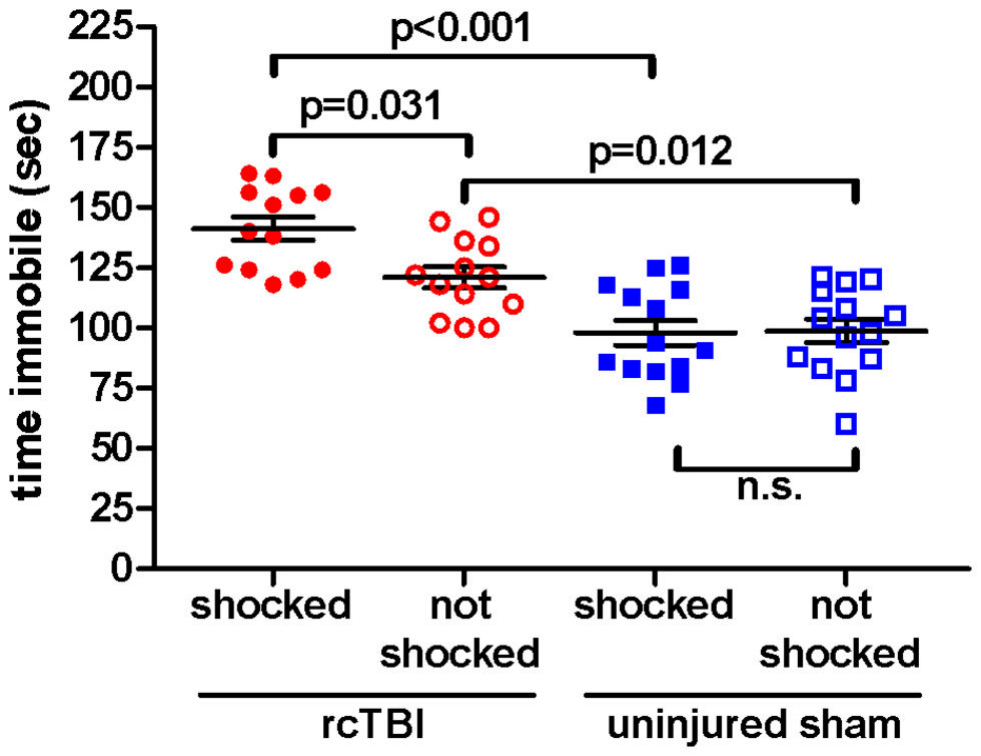
Tail Suspension Test with previously cued fear conditioned mice. Time spent immobile during a 6 minute test. Two-way ANOVA shows a significant main effect of injury (p<0.001), shock (p=0.048), and a significant interaction between injury and shock (p=0.033). Bonferroni post hoc testing shows a significant difference between immobility time in rcTBI, shocked and rcTBI, not shocked (p=0.031), rcTBI, shocked and sham, shocked (p<0.001), and rcTBI, not shocked and sham, not shocked (p=0.012), but no difference between sham, shocked and sham, not shocked (p=1.000, n.s.). Error bars represent SEM.

Time spent immobile out of the 6 minute tail suspension test was analyzed in a two-way ANOVA (Figure 6). There were significant main effects of injury [F(1, 50)=46.45, p<0.001] and shock [F(1, 50)=4.1, p=0.048], as well as a significant interaction between injury and shock [F(1, 50)=4.79, p=0.033]. Bonferroni post hoc tests indicate significant differences between immobility time in rcTBI, shocked vs. rcTBI, not shocked (p=0.031); rcTBI, shocked vs. sham, shocked (p<0.001); and rcTBI, not shocked vs, sham, not shocked (p=0.012); but no difference between sham, shocked vs. sham, not shocked (p=1.000, n.s.).

**Figure 7:**
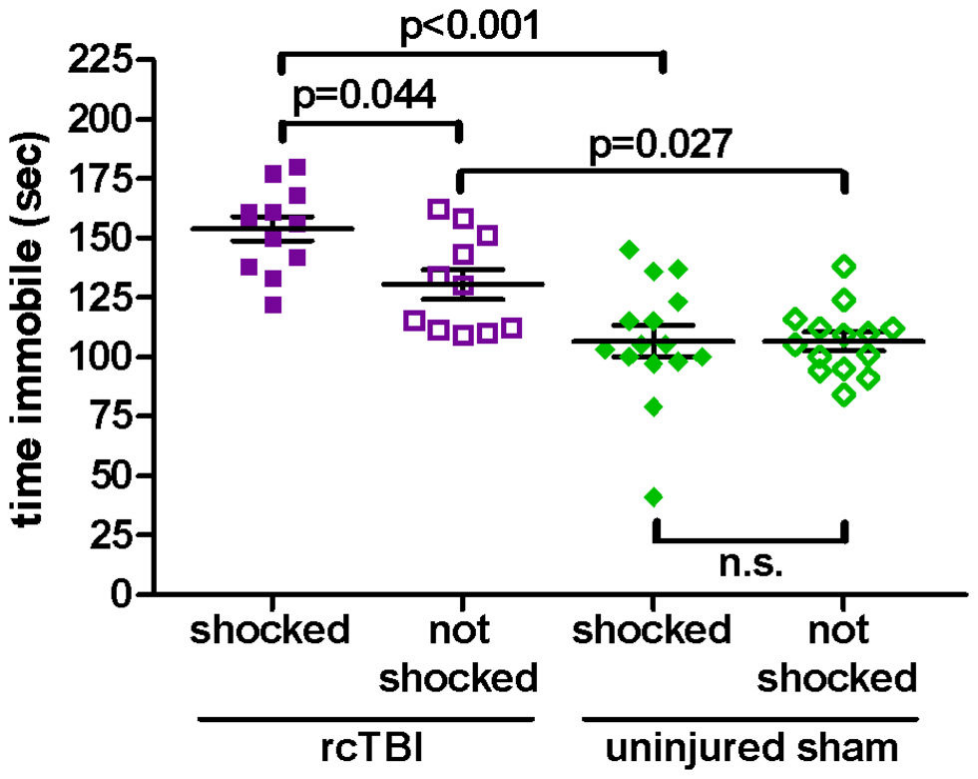
Tail Suspension Test with previously context fear conditioned mice. Time spent immobile during a 6 minute test. Two-way ANOVA shows a significant main effect of injury (p<0.001), shock (p=0.041), and a significant interaction between injury and shock (p=0.042). Bonferroni post hoc testing shows a significant difference between immobility time in rcTBI, shocked and rcTBI, not shocked (p=0.044), rcTBI, shocked and sham, shocked (p<0.001), and rcTBI, not shocked and sham, not shocked (p=0.027), but no difference between sham, shocked and sham, not shocked (p=1.000, n.s.). Error bars represent SEM.

The same analysis was applied to the group of context conditioned animals as was carried out for the cued conditioning group reported above (Figure 7). Two-way ANOVA shows significant main effects of injury [F(1, 48)=40.63, p<0.001] and shock [F(1, 48)=4.42, p=0.041], as well as a significant interaction between injury and shock [F(1, 48)=4.34, p=0.042]. Bonferroni post hoc tests indicate significant differences between immobility time in rcTBI, shocked vs. rcTBI, not shocked (p=0.044), rcTBI, shocked vs. sham, shocked (p<0.001), and rcTBI, not shocked vs. sham, not shocked (p=0.027), but no difference between sham, shocked vs. sham, not shocked (p=1.000, n.s.). These results independently replicate the findings with the previous group.

### rcTBI Decreases Sucrose Preference Independently of Prior Shock Stress in the Context Conditioned Group

**Figure 8:**
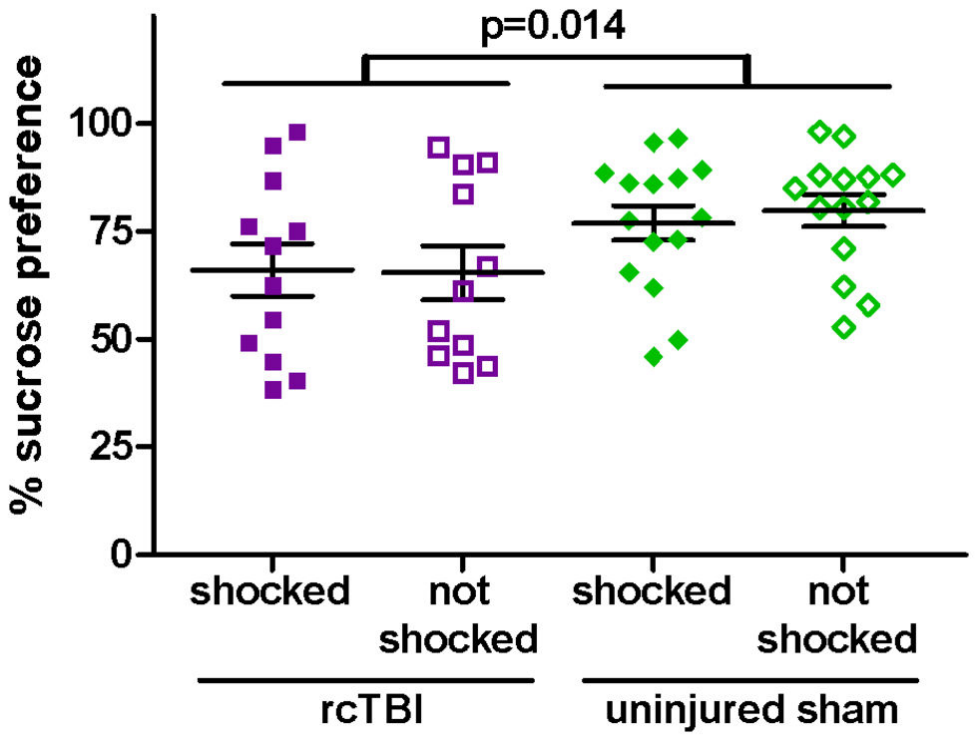
Sucrose Preference Test with previously context fear conditioned mice. Percent preference for sucrose over water during 24 hours. Two-way ANOVA shows a significant main effect of injury (p=0.014) but no main effect of shock (p=0.818, n.s.). Error bars represent SEM.

Percentage preference for 1% sucrose vs. tap water over a 24 hour period was analyzed in a two-way ANOVA (Figure 8). There was a significant main effect of injury [F(1, 50)=6.516, p=0.014], but no main effect of shock [F(1, 50)=0.053, p=0.818]. The injury x shock interaction term was non-significant [F(1, 50)=0.130, p=0.720), and Bonferroni post-hoc testing revealed no significant difference between rcTBI, shocked vs. sham, shocked (p=0.722) or rcTBI, not shocked vs. sham, not shocked (p=0.294).

### rcTBI Elevates Iba1-Positive Activated Microglia in the Corpus Callosum Independently of Shock Stress

**Figure 9:**
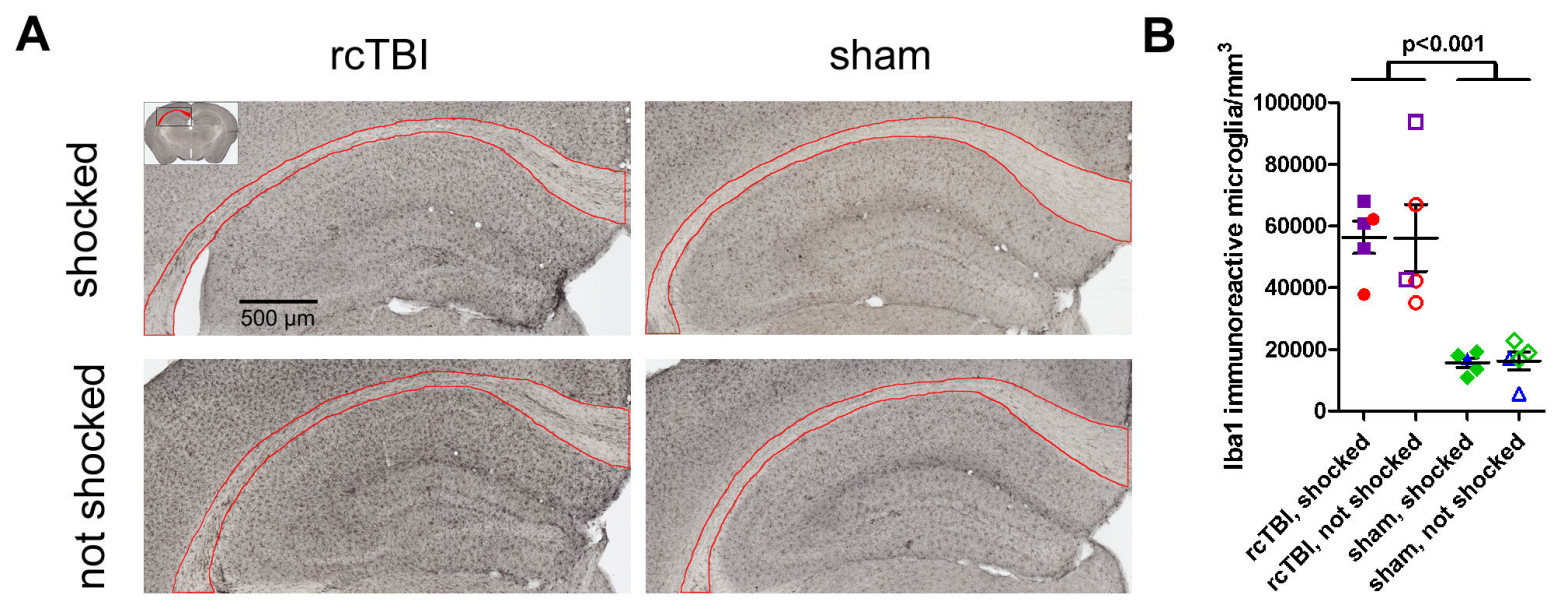
Microglial activation 40 days after injury. A) Iba1 positive cells in the corpus callosum of representative examples of mice from the rcTBI, shocked; rcTBI, not shocked; sham, shocked; and sham, not shocked groups. There is an increase in Iba1 positive activated microglia in the corpus callosum of rcTBI injured mice regardless of shock status. Boundaries of the ipsilateral corpus callosum for stereological counts outlined in red. B) Stereological quantification of Iba1 immunoreactive microglia in the corpus callosum ipsilateral to the rcTBI injury. Points are colored to correspond with earlier figures (red and blue points: cued conditioning group; purple and green points: contextual conditioning group). Error bars represent SEM. 2-way ANOVA shows a significant main effect of injury (p<0.001), but no significant main effect of shock (p=0.984, n.s.) or interaction of injury and shock (p=0.953, n.s.).

Iba1 immunoreactive microglia were quantified in the corpus callosum ipsilateral to the rcTBI or sham injury site using the Stereoinvestigator optical fractionator (Figure 9). Two-way ANOVA analysis showed a significant main effect of injury [F(1,19)=41.6, p<0.001], but no significant main effect of shock [F(1, 19)=0.0004, p=0.984] or interaction between injury and shock [F(1,19)=0.0036, p=0.953]. The Gunderson coefficient of error was ≤0.1 for all counts.

## Discussion

### Summary

We investigated hedonic, depression-like, and social behaviors in mice after repetitive concussive traumatic brain injury followed by foot shock stress. We hypothesized that rcTBI injured mice would have altered fear learning and extinction in cued and contextual fear conditioning. Contrary to our hypothesis, there was no significant difference in cued or contextual fear conditioning between the rcTBI and sham groups of mice, and no difference in the extinction of cued or contextual fear conditioning over 4 days of testing.

We subsequently tested the mice that underwent cued and contextual shock fear conditioning in tests of social and emotional behavior. We hypothesized that shock stress from fear conditioning would worsen social deficits, depression-like behavior, and anhedonia. In the social recognition test, injured non-stressed (rcTBI, not shocked) animals had normal habituation to an unfamiliar stimulus mouse over nine sessions, but interacted with a novel stimulus mouse in session 10 significantly less than sham animals. In comparison, injured stressed (rcTBI, shocked) animals showed decreased interaction with an unfamiliar stimulus mouse over nine sessions in addition to decreased interaction with the novel stimulus mouse in the final session. Shock stress alone without rcTBI (sham, shocked) did not result in any significant difference from the sham, not shocked control group. In the tail suspension test, rcTBI injury increased immobility, and congruent with our hypothesis, injured stressed (rcTBI, shocked) animals had greater immobility than injured non-stressed (rcTBI, not shocked) animals. Finally, in a 24-hour sucrose preference test rcTBI injured mice had decreased preference for sucrose (increased anhedonia), but contrary to our hypothesis, post-injury shock stress did not affect sucrose preference. These behavioral testing results were obtained in a blinded fashion with respect to injury and shock stress status.

In mice 40 days post-injury, we found elevated levels of activated microglia in the corpus callosum as assessed by Iba1 immunoreactivity. The corpus callosum carries interhemispheric connections that are important for the processing of social and emotional information in humans [38,39], and loss of the corpus callosum in mice impacts emotional memory more than object memory [40]. Iba1 positive cells were elevated in the corpus callosum of rcTBI injured animals compared to shams, regardless of their shock stress status. Iba1 immunoreactivity was previously described in this rcTBI model as an indirect marker of white matter injury as early as 3-7 days after injury, persisting until at least 49 days post-injury [26]. Comparing Iba1 counts across groups in this study, the lack of significant difference in Iba1 positive cells between the rcTBI, shocked and rcTBI, not shocked groups suggests that the differences in behavior between these two groups are not due to differences in injury severity. However, in the present cohort of mice, we found 50% higher Iba1 immunoreactivity compared to the animals assessed at 29 and 49 days post-injury in the previous study. The animals in the current study underwent several behavioral tests and much more handling than the animals in the previous study, and we hypothesize that this may have led to the observed increase in activated microglia in the present study. Direct comparisons between extensively handled and non-handled mice after injury will be required to address this.

In summary, rcTBI did not alter cued or contextual fear conditioning or extinction, and foot shock stress from fear conditioning did not worsen hedonic deficits of rcTBI injured mice in the sucrose preference test. However, post-injury shock stress worsened social interaction in both the habituation and novelty phases of the social recognition test, and increased immobility in the tail suspension test beyond the deficits seen in non-stressed injured mice (Table 2).

**Table 2:** Social and emotional behavioral phenotype of rcTBI injury model.

**behavior**	**models**	**affected by rcTBI**	**interacts with shock stress**
cued fear conditioning/extinction	fear learning/expression	no	−
context fear conditioning/extinction	fear learning/expression	no	−
sucrose preference test	anhedonia	yes	no
social recognition test	social interaction	yes	yes
tail suspension test	depression	yes	yes

Our model of rcTBI does not affect fear learning/expression in cued or contextual fear conditioning. However, prior participation in fear conditioning, including experiencing aversive inescapable foot shock stress during the training protocol, worsens subsequent injury-related impairments in social interaction and depression-like behavior in the social recognition and tail suspension tests, respectively. In contrast, prior experience with foot shock stress during fear conditioning did not further worsen injury-related anhedonia-like behavior in the sucrose preference test.

### Scientific Relevance

These findings are important because, to our knowledge, no previous studies in animal models have addressed the relationship between repetitive concussive TBI and environmental stressors. Post-injury stress appears to play a large role in negative outcomes after TBI in humans, and to date there are no consistent evidence-based guidelines for treatment of emotional and social deficits in brain injured patients, apart from treatment guidelines used in the non-injured population [8,41]. It is unknown whether the biological basis of depression, anxiety, and social impairment in the context of brain injury is the same as in the general population [42]. Animal models exploring this issue may help to resolve this question and subsequently suggest improved or alternative treatments for the brain-injured patient population. Strengths of this study include the development of a reliable animal model of the interaction between repetitive concussive brain injury and post-injury stress, careful attention to blinding and randomization, and quantitative assessments of social deficits and depressive-like behaviors that were generally reproducible across two independent experiments.

### Relationship to Previous Literature

Our fear conditioning findings in rcTBI mice differ from those reported in a model of repeated blast TBI in rats, which included increased freezing to a single presentation of a tone two days after fear conditioning [25]. However, there were many differences between these experiments (including species, timing, conditioning protocol, and type/frequency of brain injury). Fear conditioning is of great interest as a model of fear learning in PTSD research [43], and further characterization of cued and contextual fear conditioning and extinction in a variety of brain injury models is warranted to better understand how brain injury might affect the acquisition, expression, and extinction of learned fear.

In a mouse model of blast injury, results from a similar social recognition test with habituation and novelty components showed that 1 week after blast injury, C57Bl/6J mice failed to habituate to the stimulus mouse, but that this deficit resolved 2 weeks after injury [44]. Our rcTBI, not shocked mice had normal interaction with and habituation to an unfamiliar social stimulus, but decreased interaction with a second novel social stimulus mouse after nine prior presentations of the habituation stimulus mouse. This is overall in agreement with the results in the blast-injured mice, as our testing was performed at an intermediate time point: 1.5 weeks after injury. However, the effects of additional stress were not assessed in the blast injured mice, whereas in our experiments rcTBI, shocked mice also had a normal habituation curve, but less interaction with both the first and second stimulus mice compared to rcTBI, not shocked mice. Thus, in our model, the addition of shock stress after rcTBI injury resulted in a more profound impairment of social interaction than rcTBI alone.

The pattern of normal social habituation but abnormal social novelty response in rcTBI is also distinct from the social recognition impairments recognized in mice lacking oxytocin, estrogen receptors, and vasopressin receptors [28,45,46], and mice socially isolated during adolescence [47], where the experimental mice failed to habituate to the stimulus mouse. However, our findings are similar to those of Fgf17 knockout mice, which have normal social habituation but decreased response to novel stimulus mice [29]. Fgf17 is a fibroblast growth factor that contributes to patterning of the rodent frontal cortex [48]. Fgf17 knockout mice have broadly normal behavior, with the exception of specific social interactions including pup vocalization, social recognition, and social interaction while exploring a novel environment [29]. Because Fgf17 affects development of the rodent prefrontal cortex in areas that might control attention and social valuation, the social recognition test deficits in the Fgf17 knockout mouse may be attributed to impaired attention and working memory in social contexts. Further testing of social and non-social working memory in the rcTBI model may help clarify if impaired attention and working memory in social contexts is also the underlying cause of this social behavioral deficit in stressed rcTBI mice.

Our findings of increased immobility in the tail suspension test after rcTBI were consistent with a study of weight-drop TBI in Swiss mice, where the least severe injury resulted in increased immobility in the tail suspension test [49]. Likewise, a model of controlled cortical impact (CCI) TBI in mice also found increased immobility time in the tail suspension test, though in this study only the more severe injuries were associated with significant increases in immobility time [50]. The tail suspension test was originally devised to assess acute antidepressant activity of pharmacological compounds, but has also been interpreted as testing a stress response endophenotype [51]. Further investigation of both acute and chronic stress responses, particularly acute and chronic social defeat, would help to better characterize depression-like and social behavior in the rcTBI model.

In our study, we utilized painful footshock fear conditioning as a stressor, which also allowed us to examine fear learning. However, it would be interesting to assess the effects of additional types of innate, unlearned stressors after traumatic brain injury. In rodents, physical restraint [52], social isolation [53,54], predator threat exposure [55], and chronic unpredictable stress [56] paradigms can all evoke stress and avoidance responses without requiring learning. Application of these types of stressors after mild or severe TBI would help to clarify the response to different types of stress after brain injury.

### Limitations and Alternative Explanations

Limitations of the current study include the use of only a single sex and strain of mice (C57Bl/6J males), one injury model (rcTBI), and two levels of foot shock stress (4 x 0.4mA shock or 4 x 0.7mA shock, all of 0.5 sec duration). Additionally, in this study we only investigated behavior up to 18 days post-injury, with no assessment of the chronic effects of rcTBI and shock stress. For these behavior tests, we used a sample size adequate to find significant differences in behavior between groups, but our sample size was perhaps too small for the sucrose preference test, which had high variance in its dependent variable (percent sucrose preference).

In the 24 hour two-bottle test of sucrose vs. water, rcTBI injured mice had a lower preference for sucrose than uninjured sham mice, but contrary to our hypothesis, post-injury shock stress did not affect behavior in this test. Despite following a protocol that included pre-exposure to a sucrose solution to prevent neophobia and rotation of the position of the drinking bottles with respect to the light-dark cycle to prevent cage position bias in drinking behavior, we still observed wide variability in the percent preference for sucrose across all groups. However, encouragingly our control group (sham, not shocked) mean value for percent sucrose preference (79.8% +/- 3.7SEM) is consistent with recent published values for C57/Bl6J mice [57,58]. It is possible that post-injury shock does not worsen anhedonic behavior. However, it is also possible that our test was not correctly optimized to capture small differences in this behavior. Strekalova and Steinbusch suggest a criterion for anhedonia at or below 65% sucrose preference [35], and the mean percent sucrose preference for the rcTBI, shocked group was 66.1% +/- 6.0SEM, and for the rcTBI, not shocked group was 65.4 +/- 6.23SEM. Thus, one possible interpretation of our results is that rcTBI produced only borderline anhedonia-like behavior, which was not substantially worsened by shock stress. These borderline values may be the result of comparing mice that experienced a fairly mild stressor (1 day with 4 shocks of 0.4mA for cued conditioning or 0.7mA for contextual conditioning) to non-shock controls. Chronic stress paradigms previously reported to produce anhedonia-like behavior typically involve multiple weeks of aversive stimuli [59], and/or more intensely aversive stimuli, such as social defeat stress [60,61].

Our primary finding of interest was a worsening of social behavioral deficits and depression-like behavior in rcTBI injured animals that underwent foot shock stress after injury. A possible alternative explanation for these results is that the animals randomly selected for the combined injury and stress group had worse social interaction and depression-like behavior prior to injury and stress. However, repetition of behavior tests is known to influence the outcome of the later test in mice [34,35,62], so we chose not to pre-test the mice prior to assignment to injury and stress groups. In support of our results, two independent groups of mice that experienced slightly different foot shock stress intensity levels both showed similar worsening of social and depression-like behavior in response to post-injury stress.

Finally, a limitation in comparing our results to behavioral sequelae after TBI in humans is that this study examined changes in behavior only up to two and a half weeks post-injury. The timing used in this study most closely resembles the time period when acute stress disorder may be diagnosed following trauma. Acute stress disorder describes intense emotional stress reactions that occur within one month of a discrete traumatic event, such as a motor vehicle accident or assault [63]. Patients who experience acute stress disorder after an incident that includes mild traumatic brain injury are at high risk to develop long-term emotional disorders such as post-traumatic stress disorder [64]. However, the search for biological predictors of long-term emotional outcomes in patients after mild traumatic brain injury has been equivocal, indicating that participation in litigation following injury may one of the strongest predictors of chronic emotional and cognitive impairment [5]. However, the heterogeneous nature of traumatic brain injuries in patient populations might obscure links between biological injury and behavioral outcome, and further animal studies of both acute and chronic behavioral changes after brain injury may clarify these issues.

### Implications and Future Directions

In summary, we find that post-injury foot shock stress and rcTBI have synergistic negative effects in the social recognition test and the tail suspension test, but foot shock stress in the absence of rcTBI does not affect behavior in these tests. In contrast, rcTBI significantly decreases sucrose preference, but post-injury foot shock stress does not have any effect in this test. To our knowledge this is the first investigation of the interaction of post-injury stress and rcTBI with regard to emotional and social behavior in an animal model. The results of this study begin to address the role of stress in mood and social deficits in the context of concussive traumatic brain injury. Further study of the interaction of brain injury, social and non-social learning and memory, and chronic or intense stressors such as social defeat would greatly add to both our rcTBI-based model as well as other models of TBI. Most importantly, development of this model provides a new preclinical tool for testing the efficacy of standard and novel therapies for depression and social impairment in the context of concussive traumatic brain injury. Mood and social disorders play a significant part in the aftermath of concussive brain injury, and better animal models encompassing emotional and social behavior in addition to cognitive behaviors are urgently needed to develop safe and effective treatments for brain injury patients.
